# A Multidisciplinary Phenotyping and Genotyping Analysis of a Mapping Population Enables Quality to Be Combined with Yield in Rice

**DOI:** 10.3389/fmolb.2017.00032

**Published:** 2017-05-22

**Authors:** Mariafe Calingacion, Roland Mumm, Kevin Tan, Lenie Quiatchon-Baeza, Jeanaflor C. T. Concepcion, Jos A. Hageman, Sangeeta Prakash, Melissa Fitzgerald, Robert D. Hall

**Affiliations:** ^1^Grain Quality and Nutrition Centre, International Rice Research InstituteLaguna, Philippines; ^2^Laboratory of Plant Physiology, Wageningen University and ResearchWageningen, Netherlands; ^3^Wageningen Plant Research, Wageningen University and ResearchWageningen, Netherlands; ^4^Netherlands Metabolomics CentreLeiden, Netherlands; ^5^Department of Food Science and Technology, School of Agriculture and Food Sciences, University of QueenslandBrisbane, QLD, Australia; ^6^Biometris, Wageningen University and ResearchWageningen, Netherlands

**Keywords:** rice, segregating population, aroma, QTL, metabolomics, yield, GC-MS analysis, sensory

## Abstract

In this study a mapping population (F_8_) of ca 200 progeny from a cross between the commercial rice varieties Apo and IR64 has been both genotyped and phenotyped. A genotyping-by-sequencing approach was first used to identify 2,681 polymorphic SNP markers which gave dense coverage of the genome with a good distribution across all 12 chromosomes. The coefficient of parentage was also low, at 0.13, confirming that the parents are genetically distant from each other. The progeny, together with both parents, were grown under irrigated and water restricted conditions in a randomised block design. All grain was harvested to determine variation in yield across the population. The grains were then polished following standard procedures prior to performing the phenotyping analyses. A Gas Chromatography—Mass Spectrometry approach was used to determine the volatile biochemical profiles of each line and after data curation and processing, discriminatory metabolites were putatively identified based on in-house and commercial spectral libraries. These data were used to predict the potential role of these metabolites in determining differences in aroma between genotypes. A number of QTLs for yield and for individual metabolites have been identified. Following these combined multi-disciplinary analyses, it proved possible to identify a number of lines which appeared to combine the favourable aroma attributes of IR64 with the favourable (higher) yield potential of Apo. As such, these lines are excellent candidates to assess further as potential genotypes to work up into a new variety of rice which has both good yield and good quality, thus meeting the needs of both farmer and consumer alike.

## Introduction

Flavour and aroma are two of the most important factors influencing the quality of rice as perceived by the consumer. Fragrant rice, such as basmati and jasmine, are well-known for their aroma and flavour, however, other varieties of rice are also accepted or rejected on the basis of these characteristics. Consequently, aroma and flavour dictate to a great extent, consumer preference (Del Mundo and Juliano, 1981; Fitzgerald et al., 2009). Aroma is generally the major contributor to the overall flavour in all varieties of rice (Cho et al., 2014). As a consequence, the identification of compounds contributing to rice aroma, the factors affecting the aroma profile, as well as the genetic basis of its main components have been major research concerns. Such goals are not only limited to rice breeding programs but also are of general relevance to research programs on many food crops where advanced instrumentation for the detection of volatile aromatic compounds is becoming more widely used (Hall et al., 2008).

Over 100 volatile compounds have been reported to be present in rice. However, only few may be key to characterizing and defining rice aroma. In particular, odour threshold (human limit of detection), plays a major role in determining whether individual compounds contribute to the aroma phenotype (Buttery et al., 1988; Jezussek et al., 2002; Yang et al., 2010; Bryant and McClung, 2011; Calingacion et al., 2012; Mathure et al., 2014). Little is known about the genetic basis of aroma in rice, or the inheritance patterns of any of the key flavour compounds. The exception to this is 2-acetyl-1-pyrroline (2AP), which is associated with the popcorn-like/floral aroma of fragrant rices and for which the genetic background has, at least in part, been established (Bradbury et al., 2005; Chen et al., 2008; Kovach et al., 2009).

Clearly the aroma of rice is not as simple as the presence or absence of 2AP and thus is not the consequence of just a single determinant (e.g., Mumm et al., 2016). There are many other naturally-occurring compounds that are volatile, or which are products of lipid oxidation or which occur as a result of Maillard reactions upon cooking, thus contributing to complex aroma profiles comprising multiple aromatic compounds, including alcohols, alkanes, alkenes, aldehydes, and other reductones. While these compounds may only be present at low concentrations, nevertheless, many have also a low odour threshold and hence can play an influential role in detectable aroma (Demyttenaere et al., 2003). Furthermore, it is well-known that sensory panels are readily able to distinguish between the aromas of different varieties of cooked rice (Champagne et al., 2010) using descriptors of both pleasant and unpleasant notes. For example, IR64, a popular variety of rice (a so-called “mega”—variety) has been described as having a pleasant aroma, whereas Apo, a less popular variety has been described as having a number of less pleasant aroma attributes (Champagne et al., 2010).

IR64 is a variety of rice that is popular with consumers in many Asian countries (Mackill et al., 2012). Ever since its release in 1985 it has been grown annually on more than one million hectares of land (Fitzgerald et al., 2009). IR64 has been adopted by farmers and accepted by consumers, mainly due to its excellent eating quality (Champagne et al., 2010). However, IR64 is susceptible to a number of abiotic and biotic stresses which can significantly limit yield potential and entail seasonal risks to production (Venuprasad et al., 2007). In contrast, Apo is tolerant to many stresses, especially drought, and is reported to give high yield under the drought conditions characteristic of upland areas as well as in lowland, well-watered areas. This aspect of “reliable” yield is of growing importance in rice production. If we could capture the favoured agronomic and quality traits of both varieties in a single genotype, this could lead to the release of a new variety in many areas of Asia that would be valuable to farmers and consumers alike. We have previously shown that the volatile metabolomes of Apo and IR64 grains contain many metabolites that associate with the sensory descriptors of each, in both positive and negative terms, and we have developed a panel of compounds that discriminate between the aromatic quality of IR64 and Apo (Calingacion et al., 2015).

Crop improvement programs are moving with increasing rapidity toward using customized genotyping techniques for selection (Collard and Mackill, 2008). Identification of genetic markers has become significantly less complex with the rapid evolution of genotyping technologies, such as the one million SNP chip, and genotyping-by-sequencing. Methods to phenotype specifically for aroma have also been developed in recent years, with metabolomic profiling now reaching a stage where it can be reliably used as an advanced phenotyping tool for objectives such as understanding plant aroma and food flavour (Dunemann et al., 2009; Mathieu et al., 2009; Inui et al., 2013).

For this study we have used a mapping population derived from the parents IR64 and Apo, and firstly, conducted a genotyping-by-sequencing approach to characterize the progeny. This population was then used to address the following objectives: (i) screen for contrasting aroma within the progeny by metabolomic profiling using coupled gas chromatography mass spectrometry (GC-MS); (ii) identify QTLs that associate with the yield of Apo; (iii) identify QTLs that associate with the major discriminatory metabolites of low odour threshold and that may help define the flavour characteristics of each variety; and (iv) identify individual lines displaying the yield potential of Apo combined with the metabolomic profile and grain quality of IR64.

## Materials and methods

### Plant material

Apo, IR64 and 213 recombinant-inbred lines (F_5_) derived from a cross between Apo and IR64 were planted at the Experimental Station of the International Rice Research Institute (IRRI), Philippines in the dry season of 2011. The plants were carefully monitored, and a single panicle (F_6_) was harvested from each. The selected samples were then planted at IRRI in the next season for seed increase. Seeds was then carefully harvested and conserved for planting in the following dry season.

During the dry season of 2012, 150 seeds of each of the 213 lines (F_7_) from seed increase of previous season were sown as previously described (Calingacion et al., 2015). Before transplanting, inorganic fertilizer, nitrogen: phosphorus: potassium (NPK) were applied to the field at a ratio of 40:40:40 kg ha^−1^. Seedlings were transplanted in six blocks of 100 plants each in a randomised block design, with three blocks under irrigated and three blocks under water restricted conditions. One seedling per hill was planted at a spacing of 15 cm between and within rows. After transplanting, the plants were top-dressed with urea after 30 and 55 days at a level of 30:0:0 kg ha^−1^ (NPK). A small piece of leaf material was taken from one plant in the centre of each block grown under irrigation for extraction of DNA. For blocks under water restricted conditions, stress was artificially imposed by draining the field when the plants were at the maximum tillering stage so that water stress overlapped with the reproductive stage of the plant for greatest impact. Irrigated blocks, on the other hand, were maintained at a water level of ~5 cm until harvest, at which time they were drained. Mature grains from plants in all blocks were harvested (F_8_), yield was determined, and the grains were dried in an oven until a moisture content of 12–14% was reached prior to milling. Grains were dehulled (Otake FCY2 Dehusker, Oharu, Japan), polished in a paint shaker with aluminum oxide and cryo-ground (IKA A11b basic analytical mill) with liquid nitrogen. Samples were stored at −80°C until further experimentation.

### Genotyping

#### Coefficient of parentage

In order to determine the degree of diversity between the two rice varieties that were used to develop the mapping population, the coefficient of parentage (COP; Wang and Lu, 2006) was calculated between Apo and IR64 using the COP function in the International Rice Information System (IRIS) database (McLaren et al., 2005; http://irri.org/tools-and-databases/international-rice-information-system).

#### DNA preparation and SNP scans

DNA was extracted from leaf tissue of Apo, IR64, and all 213 lines of the population, using the modified CTAB DNA extraction method (Murray and Thompson, 1980). DNA in the extracts was quantified and diluted to 50 ng μL^−1^ using a Thermo Scientific Nanodrop 1000. Genotyping by sequencing was conducted in sets of 96 samples per lane using an Illumina HiSeq instrument at Cornell University (http://www.igd.cornell.edu/index.cfm/page/projects/GBS.htm). SNP calls were made using the variety Nipponbare as reference. As the confidence level of calling the heterozygote state was low, all were considered as missing data. Only 0.65% of the data points were heterozygotes. The sites were filtered at a maximum count of 170 of 213 which accounts for sites where 80% of the lines have a call and a minimum frequency of 0.25 for the minor allele. The above criteria resulted in 2,681 filtered SNPs which were used for QTL mapping. A circular archaeopteryx tree with branch length values showing all lines, including Apo and IR64 was generated using the cladogram function in Trait Analysis by the Association Evolution and Linkage (TASSEL) program (Bradbury et al., 2007).

### Metabolomic profiling of volatile compounds

#### Headspace extraction

Rice flour (1 g) of each of the samples was placed in a 10 mL glass vial and capped. Volatile compounds in the headspace were collected by solid phase microextraction (SPME) using a 65-μm polydimethylsiloxane-divinylbenzene fiber (Supelco, Bellefonte, PA, USA), as previously described (Calingacion et al., 2012; Verhoeven et al., 2012). The volatile compounds were thermally desorbed at 250°C by inserting the SPME fiber for 1 min into the GC injection port of a GC8000 instrument (Fisons Instruments, Cheshire, UK) equipped with an HP-5 column (50 m × 0.25 mm id × 1.05 μm film thickness) in splitless mode. The temperature program started at 45°C and remained at this temperature for 2 min, was then increased by 5°C min^−1^ to 250°C, which was then maintained for 5 min. Mass spectra were acquired over the range 35–400 m/z at 2.8 scans s^−1^, with electron impact ionization at 70 eV (MD800 electron impact MS, Fisons Instruments, Cheshire, UK).

### Data processing

Raw data from the GC-MS analyses were processed using MetAlign software (Lommen, 2009) to extract and align mass signals with a signal-to-noise ratio of >3. Only mass signals that were present in at least ten samples were retained for further analysis. Signal redundancy per metabolite was removed by means of clustering and mass spectra were reconstructed as previously described (Tikunov et al., 2012). Metabolites were putatively identified by matching the mass spectra of obtained metabolites against an in-house database as well as the NIST08 (www.NIST.gov) and Wiley spectral libraries, and by comparison with retention indices of reference standards published in the literature using a series of alkanes (Strehmel et al., 2008). VOCs having match factors lower than 800 and deviations of the RI of more than 30 units were usually not taken into consideration. The level of identification followed the criteria defined by the MSI standards initiative (Sumner et al., 2007). The processed, relative quantities of volatile metabolites were subjected to Principle Components Analysis (PCA) using SIMCA-P 14.0 (Umetrics AB, Umea, Sweden). Data were log-transformed (to improve normality) and pareto scaled (to put metabolites on the same scale while preserving their underlying structure). The number of significant PCs was determined by cross-validation (Eriksson et al., 2006). To determine dependence between metabolites, a correlation matrix was constructed using “cor” function and ggplot package in R (Wickham and Chang, 2016).

### Sensory evaluation of rice flour

For sensory evaluation using quantitative descriptive analysis (QDA), a subset of 27 samples was randomly selected (**Figure 5B**). Six trained panelists participated in this study. Rice samples were prepared by placing rice flour (1 g) in 20 mL screw-capped vials. These were heated in a water bath at 80°C for 10 min and presented immediately to the panelists for aroma analysis in randomised order. Panelists opened the lid of the vial carefully and evaluated the presence of 10 aroma notes using the training reference standards based on the work of Champagne (Champagne et al., 2010; Table 1). Six samples were evaluated by all panelists at each session. A total of 10 sessions were held, with a standard rice sample (commercially available, long grain, non-aromatic) included in every session, as a blind sample, to measure consistency of the panelists across the sessions. Reference standards for each attribute were also available for all sessions.

**Table 1:** **Flavour descriptions and reference used in the sensory evaluation of Apo, IR64, and 27 selected lines (modified from Champagne et al., 2010)**.

**Fragrance**	**Standard used**	**Description**
Sewer/animal	Hard-boiled egg	An immediate and distinct pungent aromatic in the flavour characterized as sulfur-like and generic animal. Animal aromatic in the flavour can sometimes be identified as “piggy.”
Grain/starchy	Flour mixture	A general term used to describe the aromatics in the flavour associated with grains such as corn, oats and wheat. It is an overall grainy impression characterized as sweet, brown, sometimes dusty, and sometimes generic nutty or starchy.
Floral	Potpourri	Aromatics associated with dried flowers, such as lilac or lavender. This aromatic is characterized as spicy floral as in an “old fashioned sachet.”
Hay-like/musty	Hay	A dry, dusty, slightly brown aroma/flavour with a possible trace of musty.
Corn	Canned creamed corn	The sweet aromatics of the combination of corn kernels, corn milk, and corn germ.
Grassy	Green beans	A dried, green, slightly earthy, slightly sweet aroma/flavour including grassy and fresh green bean aroma/flavour.
Sour/silage	Alfalfa	A sour fermented vegetation aroma/flavour, not decaying vegetation.
Sweet aromatic	Fairy floss	A sweet impression such as cotton candy, caramel, or sweet fruity that may appear in the aroma and or aromatics.
Dairy	Milk	A general term associated with aromatics of pasteurized cow's milk. Most apparent just before swallowing.
Popcorn/pandan	Popcorn	A dry, dusty, slightly toasted, and slightly sweet aromatic in the flavour that can be specifically identified as popcorn.

Prior to PCA analysis, each one of 10 sensory traits was fitted separately to a linear model using the statistical software R (R Core Team, 2014). The sensory traits were used as the response variable while the factors “assessor,” “session number,” and “sample” were included as explanatory terms. Least squares means (LSmeans) were calculated for the samples, thereby effectively correcting for differences between assessors and sessions. These LSmeans were autoscaled and summarized in a PCA biplot (SIMCA-P 14.0, Umetrics AB, Umea, Sweden).

### Marker-trait association and QTL mapping

Broad sense heritability, H, measures the proportion of the phenotypic variance due to genetic factors (Holland et al., 2003) and is calculated as:

$$\begin{array}{l} H=Vg/\left(\frac{Vg+Ve}{r}\right) \end{array}$$

where *Vg* is genotypic variance, *Ve* is error variance and *r* is number of replications. *H* was calculated for all the traits using QTL IciMapping software 4.1 (Wang et al., 2016). All the sources of variation were considered as random while estimating variance components.

After processing of the genotyping data and validating against known genes for amylose content and gelatinisation temperature (data not shown), QTL mapping could be carried out using a subset of 184 progeny and the parents Apo and IR64. The generated report and map file were taken for QTL analysis by composite interval mapping (CIM) using the QGene software V4.3.8 (Joehanes and Nelson, 2008). The genetic distances between SNP markers were estimated from the physical map based on the genomic sequence available at GRAMENE (www.gramene.org), with genetic distance (cM) = Physical distance (kb)/250. CIM was performed using the standard model with a walk speed of 2 cM. Cofactor selection was set to auto. Permutation tests were performed for each trait with composite interval mapping and 1,000 permutations (Churchill and Doerge, 1994). Marker-trait association was conducted by using the TASSEL program (Bradbury et al., 2007). The filtered sites which were polymorphic among the parents were then used for association analysis using a general linear model (GLM). In this study, only QTLs with a significance threshold of *p* < 0.0001 (−log_10_ *p* = 3.0) identified for yield under irrigation and water restricted and for discriminating metabolites were used. Genotypic and phenotypic data (Calingacion et al., 2017) were used for QTL mapping using Qgene software as described above.

## Results

### Genotyping

**Figure 1:**
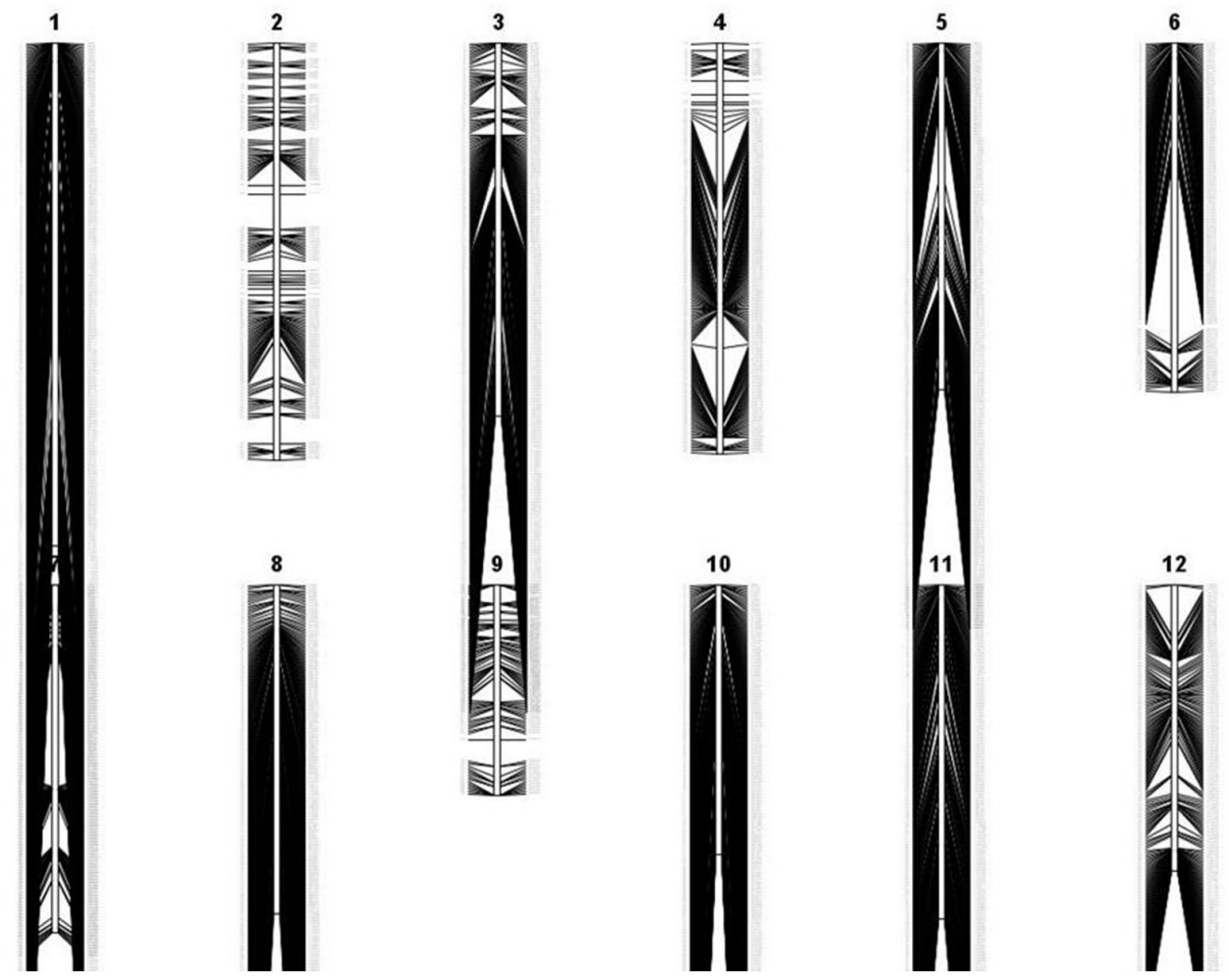
**Genetic linkage map of 2,681 polymorphic markers in F_8_ recombinant-inbred rice lines derived from Apo and IR64, generated by QGene version 4.3.8 (Joehanes and Nelson, 2008)**.

**Figure 2:**
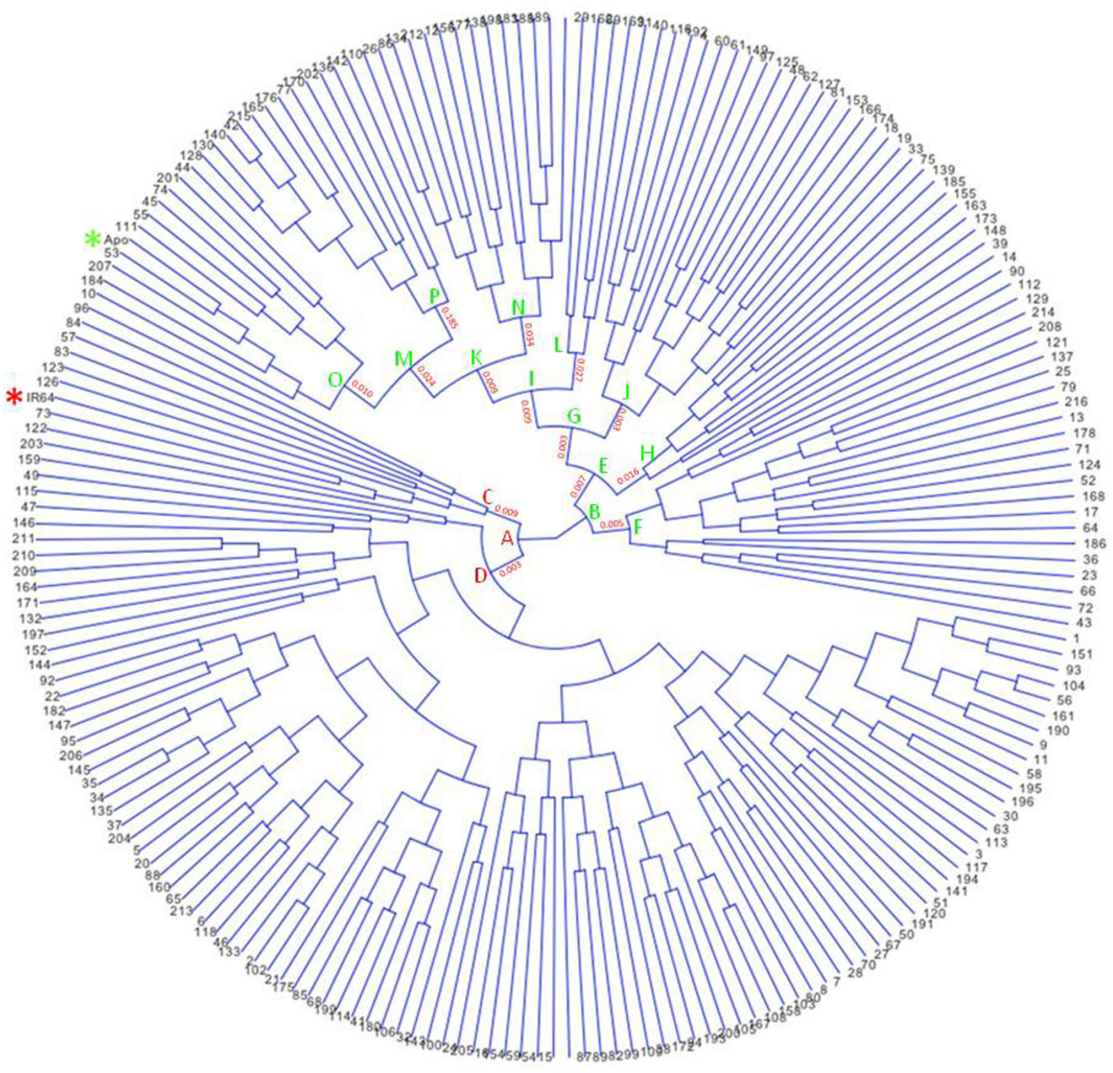
**Circular archaeopteryx tree showing 213 F8 recombinant-inbred rice lines of the cross between Apo and IR64, generated using Trait Analysis by Association Evolution and Linkage (TASSEL) program (Bradbury et al., 2007)**. Stars were included to highlight Apo (green) and IR64 (red).

Apo, IR64, and 213 RILs derived from them were genotyped using genotyping-by-sequencing (GBS), then data obtained were annotated and filtered, resulting in 2,681 polymorphic SNPs. These give dense coverage of the genome, with very few gaps seen in any of the chromosomes (Figure 1). The calculated coefficient of parentage for Apo and IR64 is 0.13. Using all the genotype data, a circular archaeopteryx tree with branch length values was constructed (**Figure 2**). This tree shows two main branches, with Apo located in Group B and IR64 in Group A. There are similar numbers of progeny in each of these main branches A and B (116 and 97). After the first cluster break, giving the branches A and B, there are several sub-clusters. IR64 was in sub-cluster C along with another eight lines, while Apo was in sub-cluster O together with another 10 lines (**Figure 2**). Both sub-clusters C and O have short branch lengths of 0.009 and 0.010, respectively indicating high genetic similarity. Twenty-nine lines were removed due to insufficient genotype data, likely to be due to poor quality DNA, thus leaving 184 genoyped lines and the two parents.

### Yield under irrigated and water restricted conditions

**Figure 3:**
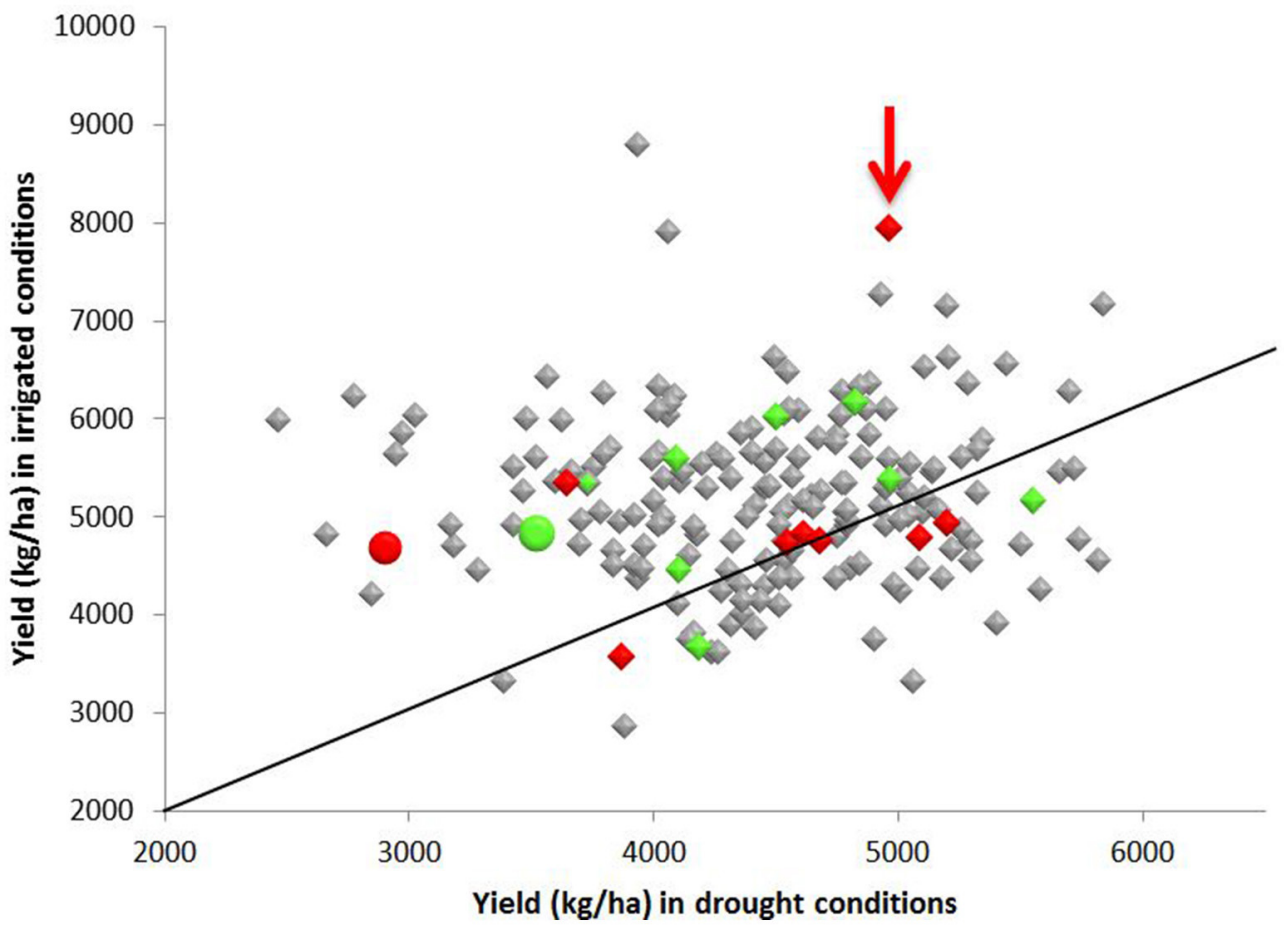
**Yield (kg ha^−1^) of 213 F8 inbred rice lines of a population grown under irrigated and water restricted conditions (Dry season 2012) at the International Rice Research Institute**. Samples of the parents, Apo (green), and IR64 (red) were also included and are shown as circles. Lines that fell within the same sub-cluster in Figure 2 as the Apo parent are highlighted in green and those falling in the same subcluster (Figure 2) as IR64 are in red. Line 83 (arrowed red) had the second highest yield under irrigation and gave high yield under water restricted conditions, and also lies in the same sub-cluster as IR64 in the archaeopteryx tree. A diagonal indicating the 1:1 ratio of yield under irrigated and water restricted conditions is also shown.

The yield of Apo under irrigated and water restricted conditions was higher than the yield obtained from IR64 grown under the same conditions (**Figure 3**). Yield of more than half of the progeny was higher than the yield of either Apo or IR64 by an average of 17% under both irrigation and water restricted conditions (**Figure 3**). Line 83 (arrowed), which is in the same genotype cluster as IR64 in Figure 2, has the second highest yield under irrigation and also gives high yield under water restricted conditions. The lines most genetically similar to either IR64 or Apo all show higher yield under water-restricted conditions than the parents, with Line 83 being the only one with a significantly higher yield under irrigation (Figure 3).

**Figure 4:**
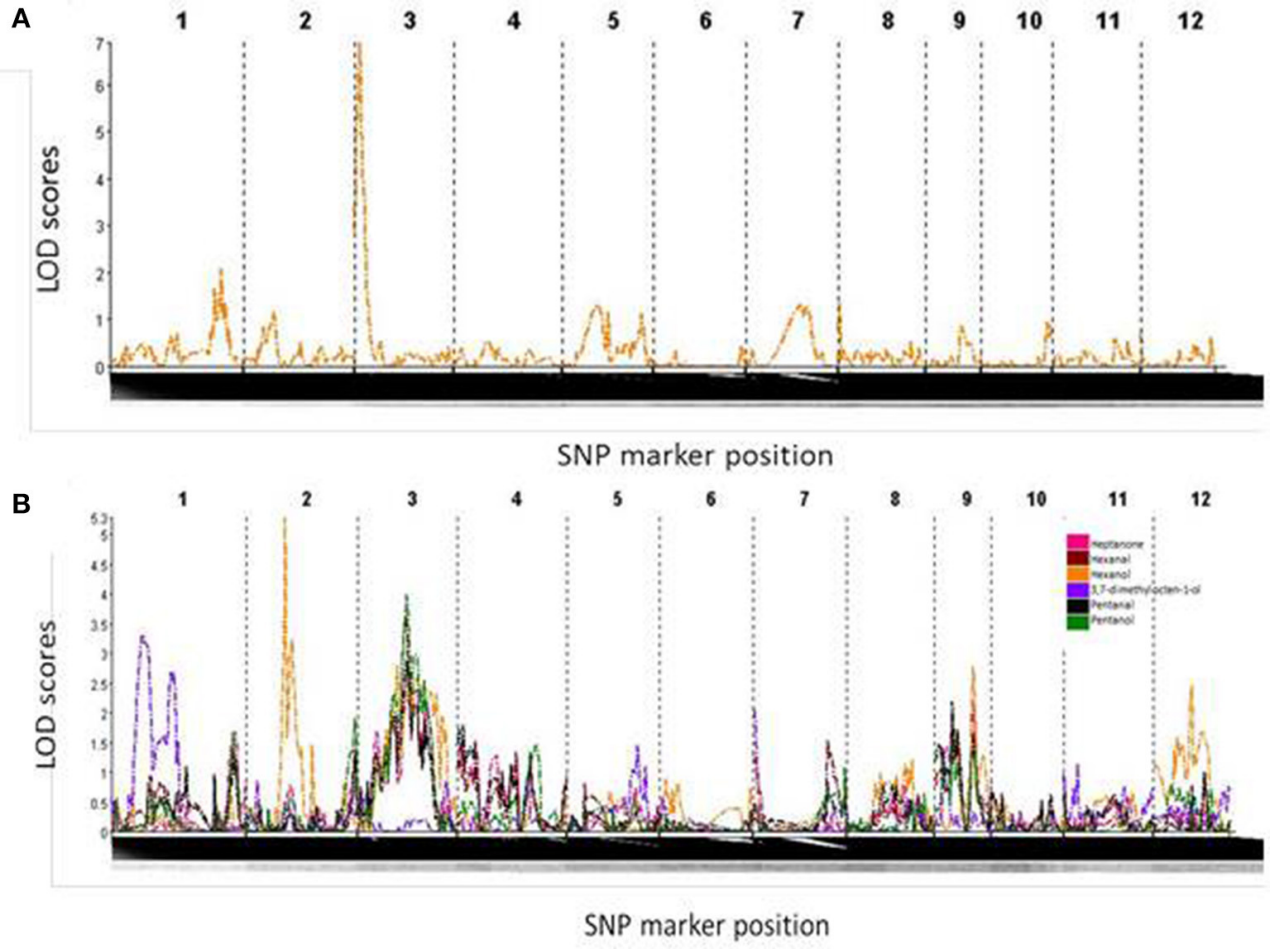
**(A)** LOD score curve denoting a strong QTL on chromosome 3 for yield under drought conditions. **(B)** LOD score curve indicating metabolite QTLs on chromosome 1 for 3,7-dimethyl,7-octen-1-ol, chromosomes 2 and 3 for hexanol, and chromosome 3 for heptanone, hexanal, pentanal and pentanol.

One significant QTL, on the short arm of chromosome 3, was found for yield under water-restricted conditions in this population (Figure 4A). This QTL spanned the interval 1.3–13.3 cM of chromosome 3, flanked by SNP markers S3_346683 and S3_3337815. The QTL peak, at SNP marker S3_1849851, was found at 7.3 cM with a LOD score of 6.814 and F statistic score of 33.8. All progeny carrying the Apo allele for this QTL had an average yield under water-restricted conditions of 4570 kg ha^−1^ which was significantly higher than the average yield of 4224 kg ha^−1^ for progeny without this QTL (*X*_2_ = 10.919, *df* = 1, *p* = 0.0009).

### Grain quality—aroma

**Figure 5:**
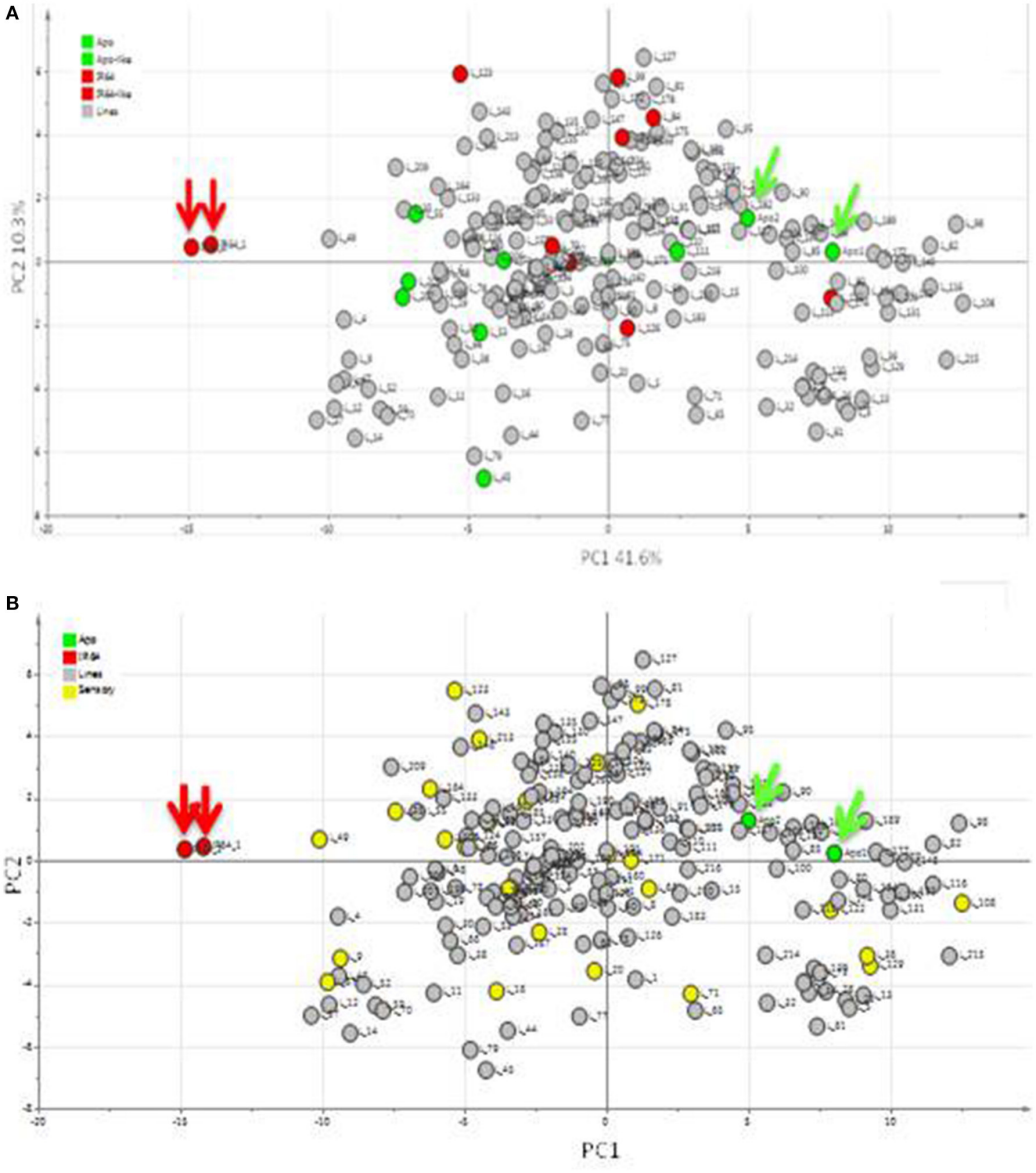
**Principal components analysis (PCA) of metabolites detected in the headspace of Apo, IR64, and 184 inbred lines derived from Apo and IR64 that were grown under irrigated conditions**. Arrows indicate the Apo (green) and IR64 (red) parental replicates. **(A)**. Score scatter plot. Lines located in the same subcluster in the archaeopteryx tree with either Apo or the IR64 as shown in Figure 2 are coloured green and red, respectively. **(B)** Same PCA score plot as in **(A)** but lines highlighted in yellow were selected for sensory analysis together with the parents (red and green).

**Figure 6:**
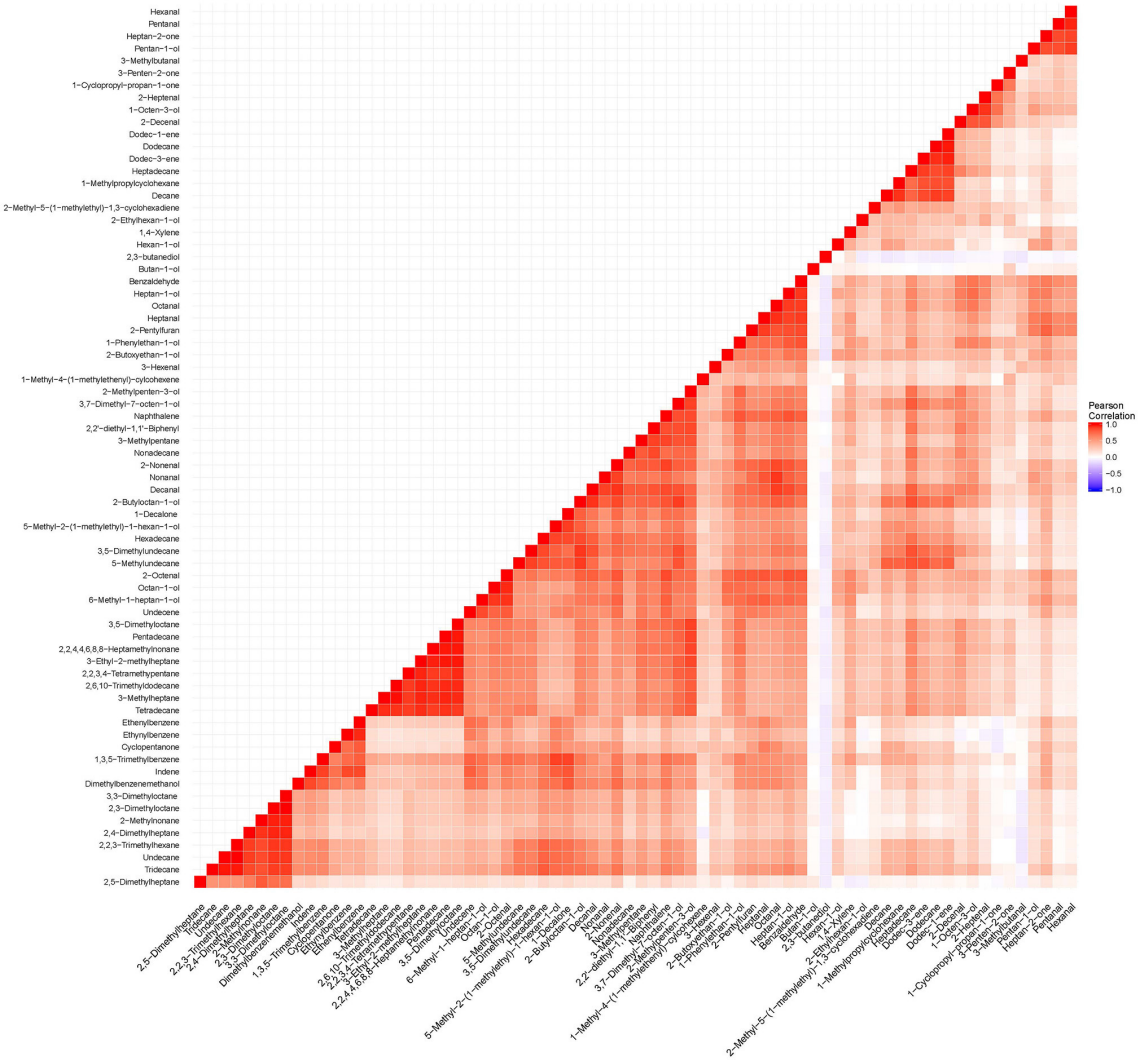
**To determine dependence between all volatile metabolites listed in Table 2, a correlation matrix was constructed using “cor” function and ggplot package in R (Wickham and Chang, 2016)**.

Using headspace sampling and GCMS, 105 compounds were detected in Apo, IR64, and 184 lines of the population (Figures 5, 6, Table 2). PC1 and PC2 together explained 55.6% of the variation in the metabolite profiles with many of the lines clustering in between the Apo and IR64 parent values. The ten lines of the population that grouped genetically with Apo in sub-cluster O and the eight that clustered genetically with IR64 in sub-cluster C (Figure 2) did not cluster in the same way based on the metabolomic profile of the grains (Figure 5A). Interestingly, most of the lines in subcluster O based on genotype data (Figure 2), showed a metabolomic profile closer to that of IR64 than Apo. Most of the lines in subcluster C with IR64 (Figure 2) showed a metabolomic profile in between those of both parents (Figure 5A).

**Table 2:** **List of putatively-identified metabolites detected in the headspace of Apo, IR64, and subset of 184 lines derived from a cross between Apo and IR64**.

**Compound**	**Aroma description**	**QTL**	**R^2^**	Additive effect^*^
**ALCOHOLS**				
Butan-1-ol	Medicinal			
Pentan-1-ol	Sweet	Chr 3 (63.3 cM)	0.093	2147.7 (Apo)
Hexan-1-ol	Grassy	Chr 2 (50.7 cM)	0.122	3582.9 (Apo)
		Chr 3 (51.3 cM)	0.066	1946.9 (Apo)
Heptan-1-ol	Citrus			
Octan-1-ol	Citrus			
1-Octen-3-ol	Mushroom			
2-Butoxyethan-1-ol				
6-Methyl-1-heptanol				
2-Ethylhexan-1-ol				
5-Methyl-2-(1-methylethyl)-hexan-1-ol				
1-Hepten-4-ol				
2-Butyloctan-1-ol				
2-Methylpenten-3-ol				
3,7-Dimethyl-7-octen-1-ol		Chr1 (41.7cM)	0.148	294.9 (IR64)
Dimethylbenzenemethanol				
2,3-butanediol	Buttery			
**ALDEHYDES**				
Pentanal	Floral			
Hexanal	Grassy	Chr3 (61.3cM)	0.086	68557 (Apo)
Heptanal	Fruity			
Octanal	Fatty			
Nonanal	Floral			
Decanal	Soapy			
Benzaldehdye	Nutty			
3-Hexenal	Leaf-like			
2-Heptenal	Fatty, grassy			
2-Octenal	Green, herby			
2-Nonenal	Fatty			
2-Decenal	Green			
3-Methylbutanal				
**KETONES**				
Heptan-2-one	Fruity	Chr3 (63.3cM)	0.067	589.7 (Apo)
Cyclopentanone				
3-Penten-2-one	Fruity to fishy			
1-Cyclopropyl-propan-1-one				
1-Decalone				
1-Phenylethan-1-ol				
**AROMATICS**				
2-Pentylfuran	Nutty, beany			
1,4-Xylene				
Indene				
1,3,5-Trimethylbenzene				
Ethenylbenzene				
1-Methyl-4-(1-methylethenyl) -cyclohexene	Citrus			
Ethynylbenzene				
Naphthalene	Mothball			
2-Methyl-5-(1-methylethyl)- 1,3-cyclohexadiene	Spicy			
**HYDROCARBONS**				
Decane				
Undecane				
Dodecane				
Tridecane				
Tetradecane				
Pentadecane				
Hexadecane				
Heptadecane				
Nonadecane				
Undecene				
Dodec-1-ene				
Dodec-3-ene				
3-Methylpentane				
3-Methylheptane				
2-Methylnonane				
5-Methylundecane				
2,4-Dimethylheptane				
2,5-Dimethlheptane				
3,5-Dimethyloctane				
2,3-Dimethyloctane				
3,3-Dimethyloctane				
3,5-Dimethylundecane				
2,8-Dimethylundecane				
2,2,3-Trimethylhexane				
2,6,10-Trimethyldodecane				
2,2,3,4-Tetramethylpentane				
2,2,4,4,6,8,8-Heptamethylnonane				
3-Ethyl-2-methylheptane				
2,2'-diethyl-1,1'-Biphenyl				
1-Methylpropylcyclohexane				

*Where known flavour attributes and QTLs were detected which are associated with the metabolites they are also indicated. The metabolites have been grouped according to their chemical classes. All putative annotations correspond with Level 2 according to the MSI standards initiative (Sumner et al., 2007)*.

* *Direction of phenotypic effect*.

Most of the compounds that were high in Apo and lines of the population that were clustered with Apo were alcohols, aldehydes, and ketones (Tables 2, 3). On the other hand, the compounds putatively identified as 2,3-butanediol and butan-1-ol were the compounds detected at high levels in both IR64 and lines of the population that were clustered with IR64 in Figure 5A, Table 2.

**Table 3:** **Descriptive statistics of compounds with known rice aroma as detected in Apo and/or IR64 and/or their recombinant inbred lines**.

**Compound**	**Apo**	**IR64**	**RILs**					
			**Mean**	**Std dev**	**Minimum**	**Median**	**Maximum**	H^*^
Butan-1-ol	2, 870.7	2, 606.7	1, 356.6	1, 564.0	359.2	1, 161.5	21, 687.2	0.94
Pentan-1-ol	36, 030.9	4, 678.5	14, 350.9	6, 322.6	4, 014.6	13, 520.2	53, 830.3	0.27
Hexan-1-ol	34, 390.7	2, 325.4	11, 054.9	7, 428.9	2, 306.5	9, 142.8	50, 848.7	0.90
Heptan-1-ol	6, 438.5	0.1	2, 218.4	1, 770.2	0.0	1, 728.5	10, 142.2	0
Octan-1-ol	62, 041.0	4, 144.5	19, 895.0	12, 769.7	4, 296.2	14, 993.2	72, 263.1	0
1-Octen-3-ol	54, 503.8	3, 134.7	38, 948.9	18, 215.0	4, 641.8	16, 361.3	115, 179.0	0
Pentanal	25, 010.0	4608.9	10240.3	5121.7	1957.3	9097.2	38, 554.5	0
Hexanal	1, 132, 634.9	165, 396.3	394, 289.4	212, 810.4	97, 915.7	352, 538.6	1, 401, 061.1	0.1
Heptanal	40, 038.6	5, 115.1	15, 157.0	8, 448.0	3, 210.3	12, 835.9	49, 618.9	0
Octanal	75, 382.1	6, 929.8	32, 431.4	22, 436.8	4, 797.9	25, 161.8	117, 040.2	0.17
Nonanal	612, 978.6	117, 179.1	273, 097.5	179, 692.4	61, 825.6	220, 074.6	1, 236, 415.9	0.74
Decanal	20, 436.0	1990.6	10, 520.9	9, 251.4	2, 128.5	7, 375.4	54, 336.3	0.92
Benzaldehyde	42, 487.4	8, 333.8	20, 187.2	11, 663.1	4, 242.0	17, 034.8	69, 762.7	0
3Hexenal	611.0	0.0	150.7	330.3	0.0	0.0	3, 660.8	0.75
2-Heptenal	24, 551.6	3, 312.4	29, 159.5	31, 992.2	1, 913.4	20, 111.6	226, 904.2	0.69
2-Octenal	15, 023.1	335.1	4, 866.4	3, 769.1	531.6	3, 571.9	22, 714.3	0.97
2-Nonenal	18, 244.4	1, 029.4	7, 690.0	8, 139.7	811.7	5, 164.8	53, 827.1	0.83
2-Decenal	540.6	0	1, 306.0	2, 213.1	0	544.4	15, 297.4	0.99
Heptan-2-one	9, 997.6	436.1	2, 683.0	2, 093.2	289.7	2, 056.7	14, 369.9	0.05
2-Pentylfuran	20, 739.9	449.4	4, 910.8	4, 342.6	219.4	3, 427.7	25, 758.0	0
3,7-Dimethyl-7-octen-1-ol	385.9	0.1	238.3	546.2	0	0	2, 619.8	1
Naphthalene	3, 162.2	111.6	2, 060.2	2, 297.8	138.2	1, 181.2	14, 576.8	0.82

* *Broad sense heritability*.

Metabolite QTLs (mQTLs) that are related to rice aroma were detected on chromosomes 1, 2, and 3 (Figure 4B). The compound which was putatively identified as 3,7-dimethyl-7-octen-1-ol was found to associate with the regions spanning the SNP markers (position) S1_5944962 (23.7 cM) and S 1_14444337 (57.7 cM). The QTL peak at chromosome 1 was mapped at 41.7 cM with an LOD score of 3.298 and *F*-statistic score of 16.121 (Figure 4B, Table 1). The compound annotated as hexan-1-ol was found to be linked to regions spanning SNP markers S2_27187174 (28.7 cM) and S2_23698655 (94.7 cM). The peak QTL for the annotated compound hexan-1-ol was found at chromosome 2 at the position 50.7 cM with LOD score of 5.283 and *F*-statistic score of 25.705. The compound annotated as hexanal associated with the regions spanning the SNP markers S3_12847023 (51.3 cM) and S3_16837834 (67.3 cM). A QTL peak at chromosome 3 was mapped at 61.3 cM with an LOD score of 3.631 and *F*-statistic score of 17.304 (*p* < 0.01; Figure 4B, Table 2). A QTL for the compound annotated as heptan-2-one was also found in the regions encompassed by the markers S3_12847023 (51.3 cM) and S3_16837834 (67.3 cM), with the QTL peak located at 63.3 cM, providing a LOD score of 2.834 and *F*-statistic score of 13.374 (Figure 4B, Table 2). The annotated compound pentan-1-ol was also mapped to the regions encompassed by the markers S3_12847023 (51.3 cM) and S3_16837834 (67.3 cM), with QTL peak located at 63.3 cM, providing a LOD score of 3.985 and *F*-statistic score of 19.078 (Figure 4B, Table 2). QTLs could not be found for either butan-1-ol or 2,3 butanediol, the two main compounds that clustered with IR64, nor several of the other compounds. The phenotype values were not always normally distributed in the population, which is an assumption of the model used for QTL mapping. Possible explanations include (i) the phenotypic distribution of the population was skewed, and/or (ii) the genetic variants associated with these metabolites are present as rare alleles in the population.

**Figure 7:**
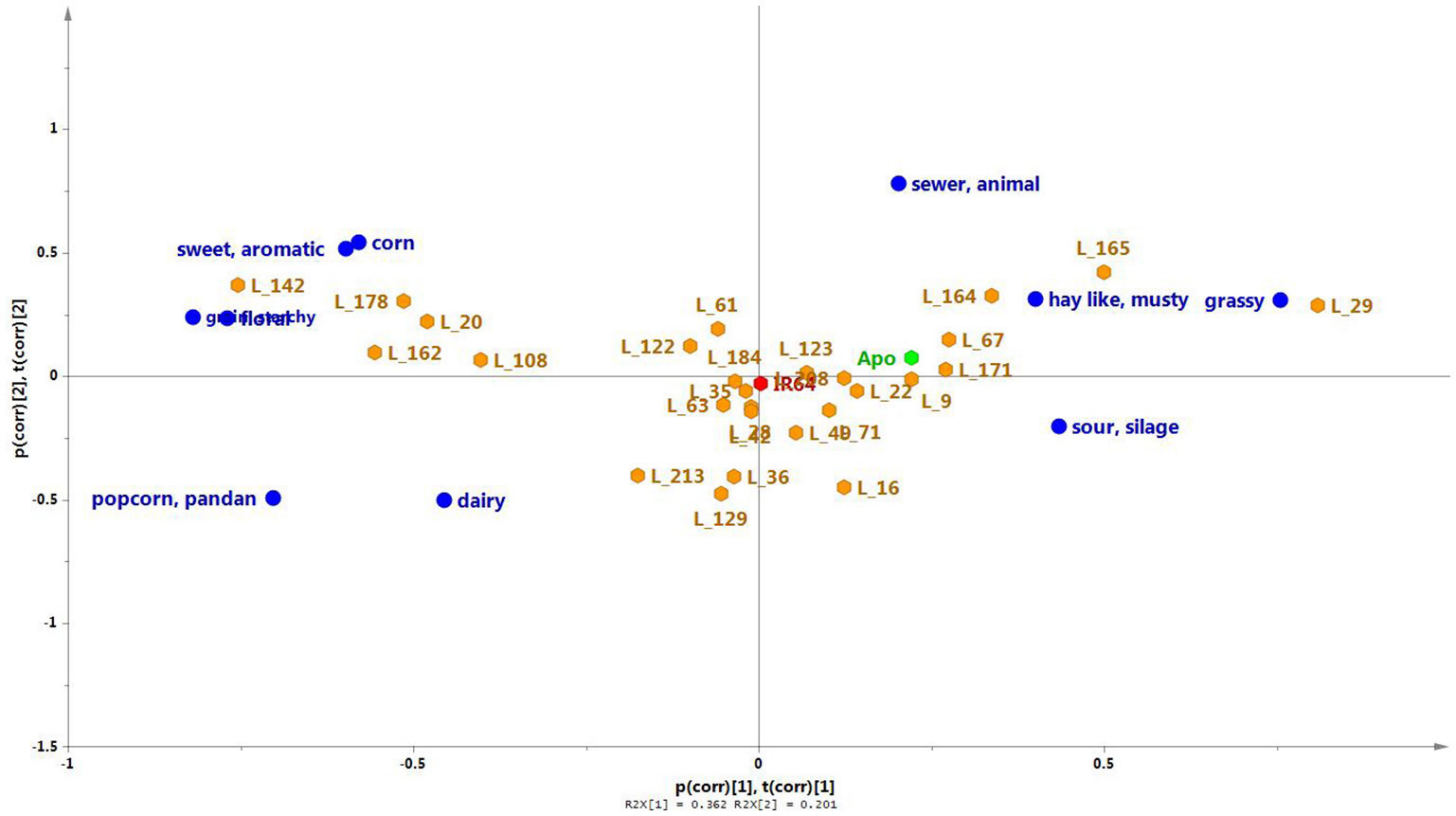
**A PCA biplot showing the scores (orange) and loadings (green) of the first two principal components of the sensory aroma evaluation of Apo, IR64, and a selection of 27 inbred lines**. PC1 and PC2, respectively, explain 36.2 and 10.1% of the total variation. The parent lines are indicated in red (IR64) or blue (Apo).

Twenty seven of the 184 lines of the population were randomly selected for sensory profiling, together with the two parent lines (Figures 5B, 7). Sensory profiling was carried out using 10 aroma attributes (Table 1). After correcting the data for differences between panelists and sessions, the data were subjected to PCA. Notes of hay-like/musty and sour silage were positively correlated with grassy notes in a PCA biplot jointly showing the correlation structure of the samples and sensory attributes (Figure 7). Along with the attribute sewer/animal, these notes are negatively correlated with more sweet and generally more pleasant notes including sweet/aromatic, corn, floral, and grain/starchy (Figure 7). The attributes popcorn/pandan and dairy form a separate group on the biplot.

Among the aroma descriptors evaluated by the sensory panel, IR64 was located at the center of the PCA plot (Figure 7). Apo, on the other hand, was described as having more of hay-like/musty, grassy, and sour silage aroma but had no floral scent. Of the progeny, several lines were observed by the panelists to have similar aroma to that of IR64, being also located in the center of the plot in Figure 7. Lines 9, 67 and 171, and 22 were observed to have similar aroma as that perceived in Apo. Interestingly, five lines (20, 108, 142, 162, 178) had high levels of typical sweet (sweet/aromatic, corn, grain/starchy) and floral aroma, while Lines 164, 165, and 29 were observed to have more aroma of grassy, sewer animal, and grainy/starchy than the other aroma descriptors evaluated. Metabolites were associated with these descriptors using least square means, and the presence of detected QTLs was determined for discriminating metabolites in the 27 lines.

## Discussion

A rice population derived from IR64 and Apo underwent genotyping by sequencing and final processing of these data revealed 2,681 polymorphic SNPs which were well-distributed across the 12 chromosomes (Figure 1). Chromosomes 8 and 10 were the most densely covered, along with the long arm of chromosome 1 and the short arms of chromosomes 6 and 11 (Figure 1). Figure 2 shows that based on the genotyping data, the progeny separate into two main clusters, with IR64 in cluster A and Apo in cluster B. The low coefficient of parentage indicates that the parents are genetically quite distant and therefore represent a significant opportunity for recombination in a mapping population (Wang and Lu, 2006). This conclusion is also supported by the results presented in Figure 3.

### Agronomy: yield under water restriction

The parents of this Apo × IR64 mapping population differ in terms of grain quality and yield under water restricted-conditions. Drought has previously been shown not to decrease yield significantly in Apo (Venuprasad et al., 2012) and this was also observed in this investigation (Figure 3). In contrast, IR64 is susceptible to drought, and in Figure 3 we can observe that the yield of IR64 halved under water restricted conditions. In Figure 3 we also observe significant transgressive segregation, whereby many of the progeny had higher yield in water restricted condition than Apo, including the ten lines clustering with Apo in sub-cluster O and the eight with IR64 in sub-cluster C (Figure 2). Line 83, which is in the same sub-cluster C as IR64 (Figure 2), is positioned in the group of highest yield under water restricted conditions, and gave the second highest yield under irrigation. Line 28, which had the highest yield under water-restricted conditions (Figure 3) is in the IR64 main cluster of the archaeopteryx tree but is positioned in sub-cluster D (Figure 2). This large amount of transgressive segregation for yield (Figure 3) suggests that recombination has occurred at many loci that govern yield and stress tolerance. Recently, several varieties of rice have been resequenced, including IR64, and over 1,000 genes associated with drought stress have been identified (Jain et al., 2014), indicating the highly multi-genic nature of drought stress, and the likelihood of different resistance mechanisms in different germplasm. Therefore, in our population there could be many loci where recombination has occurred and this could explain the high degree of transgressive segregation observed, and it would be difficult to identify loci for drought resistance if multiple mechanisms are present in the population.

Thirteen QTLs have been identified on chromosomes 1, 2, 3, and 6 in various mapping populations (Bernier et al., 2007; Dixit et al., 2014; Kumar et al., 2014). Two QTLs have been mapped onto chromosome 3 for yield under drought (Sandhu et al., 2014). The first, qDTY_3.1_, was detected in a population derived from Apo and Swarna and is located in the interval between 9.1 and 11.0 cM. The QTL peak was located at 10.0 cM and flanked by microsatellite markers RM520 (9.1 cM) and RM416 (10.0 cM). In the present study, the QTL is mapped in the interval of 1.3–13.3 cM of chromosome 3, flanked by SNP markers S3_346683 and S3_3337815, and the QTL peak is found at 7.3 cM, with a LOD score of 6.814 and *F*-statistic score of 33.8. QTL analysis in the present study identified one major QTL on the long arm of chromosome 3 (Figure 4A) and many minor QTLs. Even though the QTL peak found in this study does not fall within the interval reported by Venuprasad et al. (2012), it is likely that the dense marker coverage from GBS enabled us to locate the QTL more closely, and it is thus predicted to be the same as qDTY_3.1_. All the lines with qDTY_3.1_ showed significantly higher yield under water-restricted conditions, indicating the importance of this QTL for this trait in this population. In the future, denser genotyping studies may be able to reveal potential roles of all the minor QTLs in drought resistance.

### Quality: aroma

The aroma of rice is usually only discussed in the context of 2AP (Buttery et al., 1983). However, there are many other volatile compounds in rice, and many of these have flavour descriptors and are known to have low odour thresholds, meaning that the human nose can detect them at relatively low concentrations (Buttery et al., 1988; Jezussek et al., 2002; Laguerre et al., 2007; Yang et al., 2010; Bryant and McClung, 2011; Calingacion et al., 2012; Mathure et al., 2014; Daygon et al., 2016). The multiple detection of these compounds in different studies indicates that these compounds are phenotypically relevant. Examples of these include alcohols, alkanes, alkenes, substituted alkanes, and alkenes as well as saturated and unsaturated aldehydes. Aroma is therefore a highly complex trait unlikely to be described by one or a small number of compounds. For example, the aroma of 2AP has been described as the roasted cracker smell of baking bread (Deblander et al., 2014), but rice containing 2AP is usually described as having a “floral” aroma (Buttery and Nam, 1999; Champagne, 2008; Mathure et al., 2011). This indicates that aroma can be determined by a suite of compounds that may combine additively or synergistically and that individual compounds may contribute differently in different matrices or within different biochemical profiles.

It is well-known that different varieties of rice have distinctly different aromas, and furthermore, that the environment can have an impact on the aroma of the polished grains obtained following different (regional) cultivation conditions (Itani et al., 2004) and also as a result of seasonal fluctuations (Bergman et al., 2000; Yoshihashi et al., 2003; Champagne et al., 2005). However, in rice improvement programs, breeders have only ever been able to select qualitatively and quantitatively for 2AP, because tools were not available to facilitate selection for any other aromatic compound or aroma profile (Calingacion et al., 2014).

In this study, five metabolites were identified to be linked with QTLs found in chromosomes 1, 2, and 3. Majority of these compounds are associated with characteristic aroma. All progeny carrying the Apo allele for QTLs linked with pentan-1-ol, hexan-1-ol, hexanal and heptan-2-one were observed to have higher levels of these metabolites with phenotypic variance ranging from 6.6 to 14.8% (Tables 2, 3). As rice breeding programs are moving increasingly toward using tools of genetic selection centred on many different, complementary platforms (Chen et al., 2013; Li et al., 2013), these metabolite QTLs offers breeders useful tools of selecting for targeted aromatic traits or can be combined with other QTLs or SNPs that are associated with desirable grain and agronomic traits on a single chip to assist in selection (McCouch et al., 2010; Tung et al., 2010; Dilla et al., 2011; Hoffmann et al., 2011; Fadista and Bendixen, 2012; Thomson et al., 2012; Johnston et al., 2013; Li et al., 2013). In order to make use of customized selection chips which also include markers for rice quality, the most important traits defining this quality must first be properly described, after which a robust and relevant phenotyping tool must become available to measure variability in these traits.

The aromatic quality of rice can be measured using new metabolomic profiling techniques such as GCMS that are able to detect volatile compounds of importance to aroma (Hall, 2006), and this data can effectively be used in QTL mapping and genetic associations to identify QTLs (Keurentjes et al., 2006; Fu et al., 2009). In the current study, metabolomic profiling of the parents and progeny of the population derived from IR64 and Apo has shown that the parents are separated by a significant distance along PC1, based on the 105 volatile compounds detected (Figure 5). The progeny data generally distribute the lines between the parents. However, the 10 lines in the same sub-cluster with Apo and the eight lines close to IR64 were found not to cluster with the relevant parent in terms of the metabolomic profile of the grains (Figure 5A). This might suggest that some of the key metabolites are not direct genetic products, but for example, may occur following oxidative chemistry which takes place, post-harvest, during grain storage, and processing. Indeed, many of the compounds detected are alcohols, alkanes and aldehydes, which are known products of fatty acid oxidation (Lam and Proctor, 2003). Furthermore, many of these compounds have aroma descriptors (Table 2) and low odour thresholds suggesting that they will likely play a true role in the aroma phenotype.

The compounds that were found to discriminate IR64 and the genetically similar lines have been putatively identified on the basis of fragmentation data and retention index to be 2,3-butanediol and butan-1-ol (Table 2). 2,3-butanediol is an alcohol with a pleasant buttery and creamy aroma (Buttery and Nam, 1999). It has also been detected in black rice (Ajarayasiri and Chaiseri, 2008). Butan-1-ol, on the other hand, is described as having a malty aroma and has also been found in other rice varieties (Buttery et al., 1988). Unfortunately, in this study we were unable to identify strong QTL for 2,3-butanediol and butan-1-ol. Moreover, because of the high odour threshold of butanol (Czerny et al., 2008) and 2,3-butanediol (Buttery and Nam, 1999), these compounds are unlikely to contribute significantly to the aroma of IR64 as detected by humans.

Several compounds were detected that discriminated Apo from both IR64 and the lines that were clustering with it. These compounds were also associated with aroma descriptors and have low odour thresholds. Pentanol and heptanone have been linked to a sweet and fruity aroma. We have identified a mQTL for heptanone on chromosome 3. Moreover, hexanol and hex-3-enal are associated with a grassy and leafy-like smell.

From the sensory aroma evaluation, IR64 was located at the center of the PCA plot indicating a “balanced” aroma with respect to the 10 descriptors observed by the panelists (Figure 7). This is an interesting finding given that the volatile profile of IR64 was quite different from all other analysed varieties (Figure 5). There were two lines (L35 and L49) having similar metabolite profiles as IR64, which were also perceived to have sensory properties similar to those of IR64. On the other hand, e.g., line 122 was also perceived to have similar aroma descriptors as those that were perceived in IR64 by the panelists but had a metabolite profile more similar to that of Apo. Similarly, Lines 171, 22, and 16 that were perceived by the panelists to have similar aroma descriptors to Apo i.e., high in hay-like/musty and sour silage, were also located in the middle of the PCA (Figure 5B). It should be noted that the metabolite PCA was based on all the volatile compounds detected in the headspace of the rice sample that may or may not contribute directly to the aroma being perceived by the panelists. The aroma perceived by the panelists will be determined only by those volatile compounds with low odour thresholds.

QTLs were found for few metabolites which may suggest that many of the metabolites of rice aroma detected are products arising after harvest, or the distribution of the trait was not normally distributed in the population. Among the QTLs found, the QTL located between 51.3 and 67.3 cM is associated with pentan-1-ol, hexanal and heptan-2-one. These compounds are found to be highly correlated (Figure 6) and their correlation may suggest that variation in their amounts are genetically controlled by certain regulators (Carreno-Quintero et al., 2012). Volatile compounds like alcohols, alkanes, aldehydes, and ketones are products of oxidation of fatty acids. However, this QTL is not co-located among the putative genes reported to regulate lipoxygenases in chromosome 3 (Umate, 2011). This regions warrants further investigation by fine mapping that would narrow down the region of interest for functional validation of candidate genes that are asssociated with these QTLs.

It is of equal importance in breeding programs to achieve increased level of yield potential and premium grain quality even under stress. A number of lines could be identified in the population such as Line 49 which have similar metabolomic and sensory properties to IR64 yet have fairly similar yields to Apo. In addition, Lines 20, 142, 162, and 178 were perceived by the panelists to have high levels of corn, floral and sweet aromatic notes, and with yields under both irrigated and drought conditions similar to that of Apo under the same conditions. Interestingly, Lines 49 and 178 contain the QTL associated with yield under drought on chromosome 3. Line 28, which had the highest yield under drought and also has the QTL associated with yield under drought, was also perceived by the panelists to have similar aroma to that of IR64 and carries metabolite QTLs that have desirable aroma.

## Conclusions

This study offers valuable information for developing new varieties with specific aroma traits as desired by consumers, through marker-assisted breeding approaches, and consumer-validated phenotyping. Using a population derived from Apo and IR64, we were able to identify lines that had similar metabolomic properties to IR64 and had comparable yield values to Apo. These lines were also located in the main cluster A of the archaeopteryx tree where IR64 is also located. Importantly, some of these lines carry the QTL associated with yield under drought on chromosome 3. These lines certainly warrant further testing in multi-location trials for potential variety release.

Six novel mQTLs for volatile compounds in rice were identified. Using a highly dense genetic map, four major QTLs for the metabolites which were annotated as pentanol, hexanol, hexanal, and heptanone were mapped to the same region in chromosome 3. Further, one QTL was detected in chromosome 1 for 3,7-dimethyl-octen-1-ol and one QTL for hexanol in chromosome 2. The importance of these QTLs in influencing metabolite variation can be validated in the future using other rice varieties and populations.

## Author contributions

MC, LQ, and JC performed the field and genotype experiments. Metabolite profiling and analysis were carried out by MC, RM, and RH. MC, KT, SP, MF, and JH conducted the sensory evaluation and analysed the data. MC, MF, RM, and RH wrote the paper. All authors read and approved the final manuscript.

## Funding

Research funded by Monsanto Beachell-Borlaug International Scholarship Program, International Rice Research Institute, and University of Queensland.

### Conflict of interest statement

The authors declare that the research was conducted in the absence of any commercial or financial relationships that could be construed as a potential conflict of interest.

## Data reports

1. Genomics data is now publicly available at UQeSpace (http://espace.library.uq.edu.au/view/UQ;520970).
2. Metabolomics data will be available in Metabolights (http://www.ebi.ac.uk/metabolights/) with study identifier MTBLS501.

## References

[B1] Ajarayasiri J. Chaiseri S. (2008). Comparative study on aroma-active compounds in Thai, black and white glutinous rice varieties. Kasetsart J. Nat. Sci. 42, 715–722.

[B2] Bergman C. Delgado J. Bryant R. Grimm C. Cadwallader K. Webb B. (2000). Rapid gas chromatographic technique for quantifying 2-acetyl-1-pyrroline and hexanal in rice (*Oryza sativa, L*.). Cereal Chem. 77, 454–458. 10.1094/CCHEM.2000.77.4.454

[B3] Bernier J. Kumar A. Venuprasad R. Spaner D. Atlin G. (2007). A large-effect QTL for grain yield under reproductive-stage drought stress in upland rice. Crop Sci. 47, 507–516. 10.2135/cropsci2006.07.0495

[B4] Bradbury L. Fitzgerald T. Henry R. Jin Q. Waters D. (2005). The gene for fragrance in rice. Plant Biotechnol. J. 3, 363–370. 10.1111/j.1467-7652.2005.00131.x 17129318

[B5] Bradbury P. Zhang Z. Kroon D. Casstevens T. Ramdoss Y. Buckler E. (2007). TASSEL: software for association mapping of complex traits in diverse samples. Bioinformatics 23, 2633–2635. 10.1093/bioinformatics/btm308 17586829

[B6] Bryant R. McClung A. (2011). Volatile profiles of aromatic and non-aromatic rice cultivars using SPME/GC–MS. Food Chem. 124, 501–513. 10.1016/j.foodchem.2010.06.061

[B7] Buttery R. Ling L. Juliano B. Turnbaugh J. (1983). Cooked rice aroma and 2-acetyl-1-pyrroline. J. Agric. Food Chem. 31, 823–826. 10.1021/jf00118a036

[B8] Buttery R. Nam Y. (1999). Flavor volatiles of rice and rice products: some recent studies. Abstracts Papers Am. Chem. Soc. 218, U30–U30.

[B9] Buttery R. Turnbaugh J. Ling L. (1988). Contribution of volatiles to rice aroma. J. Agric. Food Chem. 36, 1006–1009. 10.1021/jf00083a025

[B10] Calingacion M. Boualaphanh C. Daygon V. Anacleto R. Sackville Hamilton R. Biais B. (2012). A genomics and multi-platform metabolomics approach to identify new traits of rice quality in traditional and improved varieties. Metabolomics 8, 771–783. 10.1007/s11306-011-0374-4.

[B11] Calingacion M. (2015). Empowering Breeding Programs with New Approaches to Overcome Constraints for Selecting Superior Quality Traits of Rice. Ph.D. thesis, Wageningen University and Research Centre Available online at: http://library.wur.nl/WebQuery/clc/2078705

[B12] Calingacion M. Fang L. Quiatchon-Baeza L. Mumm R. Riedel A. Hall R.. (2015). Delving deeper into technological innovations to understand differences in rice quality. Rice 8, 6. 10.1186/s12284-015-0043-8 26054242 PMC4883128

[B13] Calingacion M. Fitzgerald M. Hall R. D. Quiatchon-Baeza L. (2017). Rice Biparental Population GBS Dataset. The University of Queensland. Dataset.

[B14] Calingacion M. Laborte A. Nelson A. Resurreccion A. Concepcion J. Daygon V.. (2014). Diversity of global rice markets and the science required for consumer-targeted rice breeding. PLoS ONE 9:e85106. 10.1371/journal.pone.0085106 24454799 PMC3893639

[B15] Carreno-Quintero N. Acharjee A. Maliepaard C. Bachem C. Mumm R. Bouwmeester H.. (2012). Untargeted metabolic quantitative trait loci analyses reveal a relationship between primary metabolism and potato tuber quality. Plant Physiol. 158, 1306–1318. 10.1104/pp.111.188441 22223596 PMC3291263

[B16] Champagne E. (2008). Rice aroma and flavor: a literature review. Cereal Chem. 85, 445–454. 10.1094/CCHEM-85-4-0445

[B17] Champagne E. Bett-Garber K. Fitzgerald M. Grimm C. Lea J. Ohtsubo K. (2010). Important sensory properties differentiating premium rice varieties. Rice 3, 270–281. 10.1007/s12284-010-9057-4

[B18] Champagne E. Bett-Garber K. Thompson J. Mutters R. Grimm C. McClung A. (2005). Effects of drain and harvest dates on rice sensory and physicochemical properties. Cereal Chem. 82, 369–374. 10.1094/CC-82-0369

[B19] Chen H. He H. Zhou F. Yu H. Deng X. (2013). Development of genomics-based genotyping platforms and their applications in rice breeding. Curr. Opin. Plant Biol. 16, 247–254. 10.1016/j.pbi.2013.04.002 23706659

[B20] Chen S. Yang Y. Shi W. Ji Q. He F. Zhang Z.. (2008). Badh2, encoding betaine aldehyde dehydrogenase, inhibits the biosynthesis of 2-acetyl-1-pyrroline, a major component in rice fragrance. Plant Cell 20, 1850–1861. 10.1105/tpc.108.058917 18599581 PMC2518245

[B21] Cho S. Nuijten E. Shewfelt R. Kays S. (2014). Aroma chemistry of African *Oryza glaberrima* and *Oryza sativa* rice and their interspecific hybrids. J. Sci. Food Agric. 94, 727–735. 10.1002/jsfa.6329 23907855

[B22] Churchill G. Doerge R. (1994). Empirical threshold values for quantitative trait mapping. Genetics 138, 963–971. 7851788 10.1093/genetics/138.3.963 PMC1206241

[B23] Collard B. Mackill D. (2008). Marker-assisted selection: an approach for precision plant breeding in the twenty-first century. Philos. Trans. R. Soc. Lond. B Biol. Sci. 363, 557–572. 10.1098/rstb.2007.2170 17715053 PMC2610170

[B24] Czerny M. Christlbauer M. Christlbauer M. Fischer A. Granvogl M. Hammer M. (2008). Re-investigation on odour thresholds of key food aroma compounds and development of an aroma language based on odour qualities of defined aqueous odorant solutions. Eur. Food Res. Technol. 228, 265–273. 10.1007/s00217-008-0931-x

[B25] Daygon V. Prakash S. Calingacion M. Riedel A. Ovenden B. Snell P. (2016). Understanding the Jasmine phenotype of rice through metabolite profiling and sensory evaluation. Metabolomics 12, 63 10.1007/s11306-016-0989-6

[B26] Deblander J. Van Aeken S. Adams A. De Kimpe N. Abbaspour Tehrani K. (2014). New short and general synthesis of three key Maillard flavour compounds: 2-acetyl-1-pyrroline, 6-acetyl-1,2,3,4-tetrahydropyridine and 5-acetyl-2,3-dihydro-4H-1,4-thiazine. Food Chem. 168, 327–331. 10.1016/j.foodchem.2014.07.088 25172717

[B27] Del Mundo A. Juliano B. (1981). Consumer preference and properties of raw and cooked milled rice. J. Texture Stud. 1, 133–140. 10.1111/j.1745-4603.1981.tb01225.x

[B28] Demyttenaere J. Macura S. De Kimpe N. Verhe R. (2003). Production of pyrazines and 2-acetyl-l-pyrroline by *Bacillus cereus* strains, in Flavour Research at the Dawn of the Twenty-First Century, eds LeQuere J. L. Etievant P. X. (London: Lavoisier), 344–349.

[B29] Dilla C. J. Reveche M. Y. Carandang S. Rey J. Mojica C. De Ocampo M. (2011). SNP marker technology in the molecular breeding pipeline. Philippine J. Crop Sci. 36, 109–110.

[B30] Dixit S. Singh A. Sta Cruz M. Maturan P. Amante M. Kumar A. (2014). Multiple major QTL lead to stable yield performance of rice cultivars across varying drought intensities. BMC Genet. 15:16. 10.1186/1471-2156-15-16 24491154 PMC3933981

[B31] Dunemann F. Ulrich D. Boudichevskaia A. Grafe C. Weber W. (2009). QTL mapping of aroma compounds analysed by headspace solid-phase microextraction gas chromatography in the apple progeny ‘Discovery’ × ‘Prima’. Mol. Breed. 23, 501–521. 10.1007/s11032-008-9252-9

[B32] Eriksson L. Johansson E. Kettaneh-Wold N. Wikstrom C. Trygg J. Wold S. (2006). Multi- and Megavariate Data Analysis, 2nd Edn. Umea: Umetrics Academy.

[B33] Fadista J. Bendixen C. (2012). Genomic position mapping discrepancies of commercial SNP chips. PLoS ONE 7:e31025. 10.1371/journal.pone.0031025 22363540 PMC3281913

[B34] Fitzgerald M. McCouch S. Hall R. (2009). Not just a grain of rice: the quest for quality. Trends Plant Sci. 14, 133–139. 10.1016/j.tplants.2008.12.004 19230745

[B35] Fu J. Keurentjes J. J. B. Bouwmeester H. America T. Verstappen F. W. A. Ward J. L.. (2009). System-wide molecular evidence for phenotypic buffering in *Arabidopsis*. Nat. Genet. 41, 166–167. 10.1038/ng.308 19169256

[B36] Hall R. (2006). Plant metabolomics: from holistic hope, to hype, to hot topic. New Phytol. 169, 453–468. 10.1111/j.1469-8137.2005.01632.x 16411949

[B37] Hall R. Brouwer I. Fitzgerald M. (2008). Plant metabolomics and its potential application for human nutrition. Physiol. Plant. 132, 162–175. 10.1111/j.1399-3054.2007.00989.x 18251858

[B38] Hoffmann T. Kvale M. Hesselson S. Zhan Y. Aquino C. Cao Y.. (2011). Next generation genome-wide association tool: design and coverage of a high-throughput European-optimized SNP array. Genomics 98, 79–89. 10.1016/j.ygeno.2011.04.005 21565264 PMC3146553

[B39] Holland J. B. Nyquist W. E. Cervantes-Martinez C. T. (2003). Estimating and interpreting heritability for plant breeding: an update, in Plant Breeding Reviews, Vol. 22, ed Janick J. (John Wiley and Sons), 9–112. 10.1002/9780470650202.ch2

[B40] Inui T. Tsuchiya F. Ishimaru M. Oka K. Komura H. (2013). Different beers with different hops. Relevant compounds for their aroma characteristics. J. Agric. Food Chem. 61, 4758–4764. 10.1021/jf3053737 23627300

[B41] Itani T. Tamaki M. Hayata Y. Fushimi T. Hashizume K. (2004). Variation of 2-acetyl-1-pyrroline concentration in aromatic rice grains collected in the same region in Japan and factors affecting its concentration. Plant Prod. Sci. 7, 178–183. 10.1626/pps.7.178

[B42] Jain M. Moharana K. Shankar R. Kumari R. Garg R. (2014). Genomewide discovery of DNA polymorphisms in rice cultivars with contrasting drought and salinity stress response and their functional relevance. Plant Biotechnol. J. 12, 253–264. 10.1111/pbi.12133. 24460890

[B43] Jezussek M. Juliano B. Schieberle P. (2002). Comparison of key aroma compounds in cooked brown rice varieties based on aroma extract dilution analyses. J. Agric. Food Chem. 50, 1101–1105. 10.1021/jf0108720 11853489

[B44] Joehanes R. Nelson J. (2008). QGene 4.0, an extensible Java QTL-analysis platform. Bioinformatics 24, 2788–2789. 10.1093/bioinformatics/btn523 18940826

[B45] Johnston S. Lindqvist M. Niemelä E. Orell P. Erkinaro J. Kent M.. (2013). Fish scales and SNP chips: SNP genotyping and allele frequency estimation in individual and pooled DNA from historical samples of Atlantic salmon (*Salmo salar*). BMC Genomics 14:439. 10.1186/1471-2164-14-439 23819691 PMC3716687

[B46] Keurentjes J. J. B. Fu J. de Vos C. H. R. Lommen A. Hall R. D. Bino R. J.. (2006). The genetics of plant metabolism. Nat Genet. 38, 842–849. 10.1038/ng1815 16751770

[B47] Kovach M. Calingacion M. Fitzgerald M. McCouch S. (2009). The origin and evolution of fragrance in rice (*Oryza sativa* L.). Proc. Natl. Acad. Sci. U.S.A. 106, 14444–14449. 10.1073/pnas.0904077106 19706531 PMC2732888

[B48] Kumar A. Dixit S. Ram T. Yadaw R. Mishra K. Mandal N. (2014). Breeding high-yielding drought-tolerant rice: genetic variations and conventional and molecular approaches. J. Exp. Bot. 65, 6265–6278. 10.1093/jxb/eru363 25205576 PMC4223988

[B49] Laguerre M. Mestres C. Davrieux F. Ringuet J. Boulanger R. (2007). Rapid discrimination of scented rice by solid-phase microextraction, mass spectrometry, and multivariate analysis used as a mass sensor. J. Agric. Food Chem. 55, 1077–1083. 10.1021/jf0623992 17256955

[B50] Lam H. Proctor A. (2003). Milled rice oxidation volatiles and odour development. J. Food Sci. 68, 2676–2681. 10.1111/j.1365-2621.2003.tb05788.x

[B51] Li H. Jiang L. Youn J. Sun W. Cheng Z. Jin T.. (2013). A comprehensive genetic study reveals a crucial role of CYP90D2/D2 in regulating plant architecture in rice (*Oryza sativa*). New Phytol. 200, 1076–1088. 10.1111/nph.12427 23902579

[B52] Lommen A. (2009). MetAlign: interface-driven, versatile metabolomics tool for hyphenated full-scan mass spectrometry data preprocessing. Anal. Chem. 81, 3079–3086. 10.1021/ac900036d 19301908

[B53] Mackill D. Ismail A. Singh U. Labios R. Paris T. Donald L. (2012). Development and rapid adoption of submergence-tolerant (Sub1) rice varieties, in Advances in Agronomy, ed Sparks D. (San Diego, CA: Academic Press), 299–352.

[B54] Mathieu S. Cin V. Fei Z. Li H. Bliss P. Taylor M.. (2009). Flavour compounds in tomato fruits: identification of loci and potential pathways affecting volatile composition. J. Exp. Bot. 60, 325–337. 10.1093/jxb/ern294 19088332 PMC3071775

[B55] Mathure S. Jawali N. Thengane R. Nadaf A. (2014). Comparative quantitative analysis of headspace volatiles and their association with BADH2 marker in non-basmati scented, basmati and non-scented rice (*Oryza sativa* L.) cultivars of India. Food Chem. 142, 383–391. 10.1016/j.foodchem.2013.07.066 24001856

[B56] Mathure S. Wakte K. Jawali N. Nadaf A. (2011). Quantification of 2-acetyl-1-pyrroline and other rice aroma volatiles among Indian scented rice cultivars by HS-SPME/GC-FID. Food Anal. Methods 4, 326–333. 10.1007/s12161-010-9171-3

[B57] McCouch S. Zhao K. Wright M. Tung C. Ebana K. Thomson M. (2010). Development of genome-wide SNP assays for rice. Breed. Sci. 60, 524–535. 10.1270/jsbbs.60.524

[B58] McLaren C. Bruskiewich R. Portugal A. Cosico A. (2005). The International Rice Information System. A platform for meta-analysis of rice crop data. Plant Physiol. 139, 637–642. 10.1104/pp.105.063438 16219924 PMC1255983

[B59] Mumm R. Hageman J. A. Calingacion M. N. Vos R. C. H. Jonker H. H. Erban A.. (2016). Multi-platform metabolomics analyses of a broad collection of fragrant and non-fragrant rice varieties reveals the high complexity of grain quality characteristics. Metabolomics 12, 1–19. 10.1007/s11306-015-0925-1 26848289 PMC4723621

[B60] Murray M. Thompson W. (1980). Rapid isolation of high molecular weight plant DNA. Nucleic Acids Res. 8, 4321–4326. 10.1093/nar/8.19.4321 7433111 PMC324241

[B61] R Core Team (2014). R: A Language and Environment for Statistical Computing. Vienna: R Foundation for Statistical Computing Available online at: http://www.R-project.org/

[B62] Sandhu N. Singh A. Dixit S. Cruz M. Maturan P. Jain R.. (2014). Identification and mapping of stable QTL with main and epistasis effect on rice grain yield under upland drought stress. BMC Genet. 15:63. 10.1186/1471-2156-15-63 24885990 PMC4048250

[B63] Strehmel N. Hummel J. Erban A. Strassburg K. Kopka J. (2008). Retention index thresholds for compound matching in GC-MS metabolite profiling. J. Chromatogr. B Analyt. Technol. Biomed. Life Sci. 871, 182–190. 10.1016/j.jchromb.2008.04.042 18501684

[B64] Sumner L. Amberg A. Barrett D. Beale M. Beger R. Daykin C.. (2007). Proposed minimum reporting standards for chemical analysis. Metabolomics 3, 211–221. 10.1007/s11306-007-0082-2 24039616 PMC3772505

[B65] Thomson M. Zhao K. Wright M. McNally K. Rey J. Tung C. (2012). High-throughput single nucleotide polymorphism genotyping for breeding applications in rice using the BeadXpress platform. Mol. Breed. 29, 875–886. 10.1007/s11032-011-9663-x

[B66] Tikunov Y. Laptenok S. Hall R. Bovy A. de Vos R. (2012). MSClust: a tool for unsupervised mass spectra extraction of chromatography-mass spectrometry ion-wise aligned data. Metabolomics 8, 714–718. 10.1007/s11306-011-0368-2 22833709 PMC3397229

[B67] Tung C. Zhao K. Wright M. Ali M. Jung J. Kimball J. (2010). Development of a research platform for dissecting phenotype–genotype associations in rice (*Oryza spp*.). Rice 3, 205–217. 10.1007/s12284-010-9056-5

[B68] Umate P. (2011). Genome-wide analysis of lipoxygenase gene family in Arabidopsis and rice. Plant Signal. Behav. 6, 335–338. 10.4161/psb.6.3.13546 21336026 PMC3142411

[B69] Venuprasad R. Bool M. Quiatchon L. Atlin G. (2012). A QTL for rice grain yield in aerobic environments with large effects in three genetic backgrounds. Theor. Appl. Genet. 124, 323–332. 10.1007/s00122-011-1707-4 21938473

[B70] Venuprasad R. Lafitte H. Atlin G. (2007). Response to direct selection for grain yield under drought stress in rice Crop Sci. 47, 285–293. 10.2135/cropsci2006.03.0181

[B71] Verhoeven H. Jonker H. De Vos R. Hall R. (2012). Solid phase micro-extraction GC–MS analysis of natural volatile components in melon and rice, in Plant Metabolomics, eds Hardy N. Hall R. (New York, NY: Humana Press), 85–99. 10.1007/978-1-61779-594-7_6 22351172

[B72] Wang J. Li H. Zhang L. Meng L. (2016). Users' Manual of QTL IciMapping. Beijing: The Quantitative Genetics Group, Institute of Crop Science, Chinese Academy of Agricultural Sciences (CAAS); Mexico: Genetic Resources Program, International Maize and Wheat Improvement Center (CIMMYT).

[B73] Wang S. Lu Z. (2006). Genetic diversity among parental lines of indica hybrid rice (*Oryza sativa* L.) in China based on coefficient of parentage. Plant Breed. 125, 606–612. 10.1111/j.1439-0523.2006.01268.x

[B74] Wickham H. Chang W. (2016). ggplot2: Elegant Graphics for Data Analysis. R Package. Available online at: https://cran.r-project.org/web/packages/ggplot2/index.html

[B75] Yang D. Lee K. Kays S. (2010). Characterization and discrimination of premium-quality, waxy, and black-pigmented rice based on odour-active compounds. J. Sci. Food Agric. 90, 2595–2601. 10.1002/jsfa.4126 20718024

[B76] Yoshihashi T. Kabaki N. Nguyen T. T. H. Inatomi H. (2003). Formation of flavor compound in aromatic rice and its fluctuations with drought stress. Res. Highlights JIRCAS 2002, 32–33.

